# Strain-dependent induction of epithelial cell oncosis by *Campylobacter jejuni* is correlated with invasion ability and is independent of cytolethal distending toxin

**DOI:** 10.1099/mic.0.2006/003962-0

**Published:** 2007-09

**Authors:** Lisa D. Kalischuk, G. Douglas Inglis, Andre G. Buret

**Affiliations:** 1 Department of Biological Sciences, Inflammation Research Network, University of Calgary, Bio 336, 2500 University Drive NW, Calgary, AB T2N 1N4, Canada; 2 Agriculture and Agri-Food Canada, 5403-1st Avenue S, Lethbridge, AB T1J 4B1, Canada

## Abstract

Induction of host cell death is thought to play an important role in bacterial pathogenesis. *Campylobacter jejuni* is a prevalent cause of bacterial enteritis; however, its effects on enterocytes remain unclear. The present study indicates for the first time that *C. jejuni* induces oncotic, rather than apoptotic death of T84 enterocytes. *C. jejuni*-treated enterocytes exhibited extensive cytoplasmic vacuolation, rapid (3–6 h) loss of plasma membrane integrity (‘cytotoxicity’), loss of mitochondrial transmembrane potential, and ATP depletion. Enterocytes also exhibited increased oligonucleosomal DNA fragmentation, a feature characteristic of apoptosis. However, consistent with a non-apoptotic process, DNA fragmentation and cytotoxicity were not caspase dependent. During apoptosis, caspases mediate cleavage of poly(ADP-ribose) polymerase; however, cleavage was not observed in *C. jejuni*-treated monolayers. Cytotoxicity, ATP depletion and DNA fragmentation were not prevented by the deletion of the cytolethal distending toxin (CDT) gene, indicating that *C. jejuni* causes enterocyte oncosis via a mechanism that is CDT independent. The ability to cause oncosis was significantly decreased in a FlaAFlaB mutant (CDT^+^) that was defective in the ability to adhere and invade enterocytes. Analysis of clinical isolates revealed that oncosis was strain dependent and correlated with increased invasive ability. These observations offer new insights into the pathogenesis of *C. jejuni* infection.

## INTRODUCTION

*Campylobacter jejuni* is the most prevalent cause of human bacterial enteritis in North America [Centers for Disease Control and Prevention (http://www.cdc.gov/foodnet/); Health Canada (http://dsol-smed.hc-sc.gc.ca/dsol-med/ndis/cdise.html)]. Infected humans exhibit a range of symptoms varying from mild watery diarrhoea to severe inflammatory diarrhoea often containing blood and leukocytes (Wassenaar & Blaser, 1999). Histological examination of affected intestinal tissues typically reveals necrotic ulceration of the mucosal epithelium with crypt abscesses and inflammatory infiltration of neutrophils into the lamina propria (Wassenaar & Blaser, 1999). Tissue damage appears to be largely due to the effects of cytotoxins (Wassenaar, 1997) and/or host-cell invasion (Everest *et al.*, 1992; Russell *et al.*, 1993). Although the pathological features of campylobacteriosis have been well described, the host–pathogen interactions involved in inciting inflammation remain poorly understood.

Host cell death is conventionally divided into the morphologically and biochemically distinct processes of apoptosis or oncosis (Majno & Joris, 1995). Apoptosis is a genetically regulated, energy-dependent process that plays a critical role in the elimination of cells during development and homeostasis (Ren & Savill, 1998), but is also implicated in the pathogenesis of a variety of enteric micro-organisms (Chin *et al.*, 2002; Fiorentini *et al.*, 1998; Kim *et al.*, 1998; Zychlinsky, 1993). Excessive enterocyte death may ultimately disrupt the intestinal epithelial barrier, allowing for increased pathogen translocation and dissemination (Buret *et al.*, 2003; Chin *et al.*, 2002). Apoptosis is characterized by chromatin condensation and fragmentation, membrane blebbing and formation of apoptotic bodies with intact plasma membranes (Majno & Joris, 1995). Phagocytic disposal of the apoptotic bodies prevents release of cellular debris and subsequent inflammation (Ren & Savill, 1998). In contrast, oncosis (often referred to as ‘necrosis’) is a rapid process characterized by vacuolation, mitochondrial swelling and loss of plasma membrane integrity (Majno & Joris, 1995). The resultant leakage of cellular content into the intercellular milieu often incites an inflammatory response leading to tissue injury (Ren & Savill, 1998).

Several morphological features can be used to distinguish apoptotic from oncotic cells (Majno & Joris, 1995). During oncosis, cells rapidly lose plasma membrane integrity after the death stimulus whereas during apoptosis, plasma membrane integrity is maintained throughout the death process until the late onset of secondary necrosis. Caspases, particularly caspase-3, play a crucial role in apoptosis by effecting many of the characteristics of apoptosis (Earnshaw *et al.*, 1999). Oligonuclosomal DNA fragmentation, which is considered a hallmark of apoptosis, is initiated by caspase-activated deoxyribonulclease (CAD) upon caspase-3 cleavage of its inhibitor, ICAD (Enari *et al.*, 1998). During apoptosis, caspase-3 also cleaves the DNA repair enzyme poly(ADP-ribose) polymerase (PARP). Inactivation of PARP prevents DNA repair and conserves cellular ATP, which is required to complete the apoptotic process (Los *et al.*, 2002).

Cell viability is largely regulated at the level of mitochondria, and cell death may be initiated by factors that cause depolarization of the mitochondrial membrane. Progression to apoptotic or oncotic cell death depends in part on the effect that mitochondrial transmembrane depolarization has on cellular ATP levels (Kim *et al.*, 2003). Cellular ATP plays a pivotal role in determining whether cell death will be apoptotic or oncotic in nature (Eguchi *et al.*, 1997; Leist *et al.*, 1997). Apoptosis is an active energy-dependent process. Specifically, ATP is required for the formation of the apoptosome, which is involved in initiating the intrinsic apoptotic death cascade (Li *et al.*, 1997). If damage to the mitochondria is such that ATP levels are insufficient to complete the apoptotic process, the mode of death may be redirected towards oncosis (Eguchi *et al.*, 1997; Leist *et al.*, 1997).

The cytotoxic effects of *Campylobacter* have been ascribed to the actions of several different toxins. However, to date only the cytolethal distending toxin (CDT) has been identified through genome sequence analysis. CDT is a DNase-like toxin produced by several species of bacteria including *Campylobacter* spp. (Pickett *et al.*, 1996). This holotoxin is composed of three subunits: CdtB has homology to the family of DNase I-like nucleases and is the active subunit, and the CdtA and C subunits function in the delivery of CdtB into target cells (Lara-Tejero & Galan, 2001). CDT is translocated into the nucleus, where it induces double-stranded DNA damage. This activates the G1 and/or G2/M checkpoint response that results in cell cycle arrest, and ultimately leads to cell death via mechanisms that are poorly understood (Whitehouse *et al.*, 1998). It has been postulated that CDT plays a role in the elimination of immune cells and therefore modulates the host immune response. The effects of CDT have primarily been described for lymphocytes and monocytes, in which it generally induces apoptosis (Hickey *et al.*, 2005; Siegesmund *et al.*, 2004; Zhu *et al.*, 1999). However, CDT has also been shown recently to induce non-apoptotic death of endothelial cells (Bielaszewska *et al.*, 2005). The mechanisms responsible for *C. jejuni*-induced enterocyte death remain obscure.

A prominent feature of campylobacteriosis is acute inflammation of the intestinal mucosa. As the mode of cell death is a decisive factor in the host inflammatory response, the present study characterized the nature of *C. jejuni*-induced enterocyte death and assessed the role of CDT in this process. Further experiments sought to determine whether the induction of enterocyte death corresponded with the ability of *C. jejuni* to invade enterocytes.

## METHODS

### Epithelial cell culture.

Studies were performed using a human colonic epithelial cell line with a crypt-like phenotype, T84 cells (American Type Culture Collection). T84 cells were grown in a 1 : 1 mixture of DMEM and Ham's F-12 medium supplemented with 10 % heat-inactivated fetal bovine serum (Sigma-Aldrich) containing 200 mM l-glutamine, 100 U penicillin ml^−1^, 100 μg streptomycin ml^−1^ and 80 μg tylosin ml^−1^ (all from Sigma). Cells were incubated at 37 °C in a humidified atmosphere containing ∼5 % CO_2_. Medium was replenished every 2–3 days and confluent monolayers were passaged with 2× trypsin-EDTA (Invitrogen). Trypsinized cells were seeded at a density of 2×10^5^ cells ml^−1^ into Lab-Tek chamber slides (400 μl per well, Nalgene Nunc International) and six-well (3 ml) or 48-well (400 μl) tissue-culture-treated plates (Costar). Unless noted otherwise, cells were grown to confluence prior to inoculation. Cells were used for experiments between passages 5 and 15.

### Bacterial strains and culture conditions.

*C. jejuni* strain 81-176, a strain widely used in pathogenesis studies and originally isolated from an outbreak of diarrhoea associated with ingestion of raw bovine milk, was used throughout the study (Korlath *et al.*, 1985). *C. jejuni* strain NCTC 11168 (clinical isolate) was also used as necessary (Gaynor *et al.*, 2004). Additional *C. jejuni* strains included 23 clinical isolates obtained from patients residing within the Chinook Health Region of Southwestern Alberta who were suffering from enteritis. All isolates were presumptively identified as *C. jejuni* based on a positive hippurate test, and their identities were confirmed by PCR detection of the *mapA* gene, which is present only in *C. jejuni* (Denis *et al.*, 1999). Isolates were stored in *Brucella* broth (Difco) containing 30 % (v/v) glycerol at −80 °C. Prior to use, cultures were streaked onto Karmali agar (Oxoid) and grown microaerophilically (5 % O_2_, 10 % CO_2_, 2 % H_2_ and 83 % N_2_) at 37 °C. Inoculum was prepared by growing *C. jejuni* in Casamino yeast extract (CYE) broth (Stanfield *et al.*, 1987) containing, per litre, 30 g Casamino acids (Difco), 4 g yeast extract (Difco), 0.5 g KH_2_PO_4_, 10 mg FeSO_4_ and 2 g porcine gastric mucin (Sigma). Mucin has previously been shown to increase invasion of Hep-2 cells (De Melo & Pechere, 1988). *C. jejuni* was grown for 14–16 h at 37 °C at 100 r.p.m. in microaerophilic conditions. Bacteria were enumerated by plating tenfold serial dilutions onto Karmali agar.

### Construction and characterization of isogenic CDT mutant.

A mutant of *C. jejuni* 81-176 was constructed by deleting the entire *cdtB* gene and part of the *cdtAC* genes, and inserting a kanamycin-resistance cassette into the deletion site. Briefly, this mutant was constructed by amplifying the promoterless *cdtABC* operon from *C. jejuni* 81-176 using previously described primers P8 and P9 (Purdy *et al.*, 2000). All PCR reactions were carried out in 20 μl volumes and contained 1× reaction buffer, 0.2 mM dNTPs, 2 mM MgCl_2_, 0.5 μM of each primer and 1 U HotStar *Taq* polymerase (Qiagen). The conditions for amplification were 1 cycle at 95 °C for 15 min followed by 30 cycles of 94 °C for 30 s, 45 °C for 30 s, 72 °C for 2 min and a final extension of 10 min at 72 °C. The resulting fragment was ligated with the pGEM-T Easy vector (Promega) and used to transform *Escherichia coli* GM2163 (Dam^−^). Mutants were selected on Luria–Bertani agar (LB) containing ampicillin (100 μg ml^−1^). Plasmid DNA was extracted with the QIAprep Spin Miniprep kit (Qiagen) and digested with *Bcl*I (New England Biolabs, NEB) to generate the deletion.

A 987 bp fragment of pACYC177 containing the Tn*903* kanamycin-resistance cassette [*aph*(3′)-1a(Kn^R^)] was amplified by PCR using primers F2kanbamH1 (5′-TTGTGGATCCTCAACAAAGCCACGTTGTGT-3′) and R2kanbamH1 (5′-TTGTGGATCCTCCCGTCAAGTCAGCGTAAT-3′) (*Bam*HI restriction site underlined). The conditions for amplification were 1 cycle at 95 °C for 15 min followed by 30 cycles of 94 °C for 30 s, 50 °C for 30 s, 72 °C for 1.5 min and a final extension of 10 min at 72 °C. The resulting fragment was ligated with the pGEM-T Easy vector and used to transform *E. coli* DH5*α* (Invitrogen). Mutants were selected on LB agar containing kanamycin (50 μg ml^−1^). Plasmid DNA was restriction-digested with *Bam*HI (NEB) and the fragment containing the Kn^R^ cassette was purified using a Wizard SV gel and PCR clean-up kit (Promega).

The *Bam*HI-digested fragment containing the Kn^R^ cassette was ligated with the *Bcl*I-digested plasmid containing the *cdt* genes, and used to transform *E. coli* DH5*α*. This construct was mobilized into *C. jejuni* using pGEM-T Easy vector (Promega) as it contains an *E. coli* origin of replication and cannot replicate in *Campylobacter*. Mutants that are kanamycin resistant arise as a result of a double homologous recombination event, in which the wild-type *cdt* operon is replaced with the *cdt* deletion containing the Kn^R^ cassette. *C. jejuni* 81-176 was transformed by electroporation as previously described (Miller *et al.*, 1988). Briefly, *C. jejuni* was grown in Columbia broth (Difco). Early-exponential-phase cells were rinsed twice with electroporation buffer (EPB: 272 mM sucrose, 15 %, v/v, glycerol, 2.43 mM K_2_HPO_4_, 0.57 mM KH_2_PO_4_). *C. jejuni* was resuspended in EPB and incubated in a 0.1 cm cuvette for 10 min (4 °C) with plasmid DNA (0.5 μg). A high-voltage pulse (12.5 kV cm^−1^) was delivered using an Electroporator 2510 (Brinkmann). Cells were immediately transferred to Karmali agar and allowed to recover for 4 h (37 °C, microaerophilic atmosphere). Cells were then harvested and plated onto Karmali agar containing kanamycin (30 μg ml^−1^). Transformed colonies were screened by PCR analysis to confirm the deletion of *cdtB* and insertion of the Kn^R^ cassette (Purdy *et al.*, 2000).

The phenotype of the CDT mutant was confirmed by inoculating T84 cells grown on chamber slides (70 % confluent) with strain 81-176 or the CDT-deletion mutant at a m.o.i. of 10. Slides treated with sterile growth medium served as controls. After 30 h incubation, cells were fixed overnight with 2 % (w/v) paraformaldehyde and stained with Hoechst 33258 (1 μM, Molecular Probes) for 25 min. Monolayers were rinsed once with phosphate-buffered saline (PBS). Slides were visualized by epifluorescent microscopy. Nuclei were enumerated and average nucleus area was determined by image analysis using Image Pro Plus v.4.1 software (Media Cybernetics). The patches of the monolayer where cells were obviously damaged and detached from the slide are referred to as ‘death foci’.

### Construction and characterization of the isogenic FlaAFlaB mutant.

The presence of a functional flagellar apparatus has been previously shown to be required for maximal invasion of epithelial cells (Konkel *et al.*, 2004). A non-invasive flagellar mutant of *C. jejuni* NCTC 11168 was constructed by deleting part of the *flaA* and *flaB* genes and inserting a Kn^R^ cassette into the deletion site. The primers used to generate the mutant did not amplify the *flaA* and *flaB* genes from *C. jejuni* 81-176, necessitating the use of the sequenced strain NCTC 11168. The mutant was constructed by amplifying the *flaA* and *flaB* genes from *C. jejuni* 11168 using primers flaAflaBF (5′-TGCTAAAGCAAACGCTGATT-3′) and flaAflaBR (5′-CTGATTGCGCAAGGATGTTA-3′). PCR amplification was carried out as described above, except that the annealing temperature was 54 °C and the extension cycle was 3.5 min. The resulting fragment was ligated with the pGEM-T Easy vector (Promega) and used to transform *E. coli* DH5*α*. Mutants were selected on LB agar containing ampicillin (100 μg ml^−1^). Plasmid DNA was extracted and digested with *EcoRV* to generate the deletion.

A Kn^R^ cassette was amplified, cloned and purified as described above except that the primers contained an *Eco*RV restriction site in place of the *Bam*HI site. The *Eco*RV-digested Kn^R^ cassette was ligated with the *Eco*RV-digested plasmid containing the *flaA flaB* genes, and used to transform *E. coli* DH5*α*. This construct was mobilized into *C. jejuni* NCTC 11168 as described above. Transformed colonies were screened by PCR analysis to confirm the partial deletion of *flaA flaB* and insertion of the Kn^R^ cassette. Motility was also examined by phase-contrast microscopy and by assessing swarming on motility agar (Columbia agar containing 0.4 % agar). The low percentage of agar allows motile bacteria to move within the agar forming a halo of growth.

### Inoculation protocol.

Confluent monolayers were rinsed twice with pre-warmed (37 °C) Dulbecco's phosphate buffered saline (PBS) (Sigma) and antibiotic-free, phenol-red-free culture medium was added to each well. Monolayers were inoculated with *C. jejuni* (cultured as described above) to achieve a m.o.i. of 100 c.f.u. per enterocyte. Control monolayers received an equivalent volume of sterile growth medium. The topoisomerase-I inhibitor camptothecin (4 μg ml^−1^ in DMSO vehicle, Sigma) and the oxidant H_2_O_2_ (0.5 mM, Sigma) were used as induction control treatments for apoptosis and oncosis, respectively (Kiechle & Zhang, 2002; Palomba *et al.*, 1999). To determine the possible involvement of caspases in *C. jejuni*-induced cell death, T84 monolayers were pre-treated with the pan-caspase inhibitor Z-VAD-FMK (120 μM in DMSO vehicle, Calbiochem) for 1 h (37 °C, 5 % CO_2_) prior to inoculation with *C. jejuni*, and inhibitors remained present throughout the *C. jejuni* challenge as described previously (Chin *et al.*, 2003).

### Oligonucleosomal DNA fragmentation assay.

Oligonucleosomal DNA fragmentation was quantified using a Cell Death Detection ELISA kit (Roche Molecular Biochemicals). This immunoassay detects histone–DNA complexed fragments (oligonucleosomes) present in the cytoplasm of apoptotic cells. Resultant absorbance values are proportional to the amount of oligonucleosomes present in the cytoplasm of cell lysates. T84 monolayers were grown in 48-well plates and inoculated with *C. jejuni* or sterile broth as described above. After challenge, medium was aspirated and oligonucleosomal DNA fragmentation in cell lysates was determined according to the manufacturer's protocol. DNA fragmentation is expressed as *A*_405_ units. According to the manufacturer, the detection limit for this ELISA is 10^2^ apoptotic cells.

### Cytotoxicity assay.

The release of the cytoplasmic enzyme lactate dehydrogenase (LDH) into the surrounding culture medium is an indicator of plasma membrane disruption (Brennan & Cookson, 2000). T84 monolayers were grown in 48-well plates and inoculated with *C. jejuni* or sterile broth as described above. Culture medium was analysed for the presence of LDH using a Cytotoxicity Detection kit (Roche Molecular Biochemicals) according to the manufacturer's protocol. Total and spontaneously released LDH activity was also determined. Results are presented as percentage cytotoxcity, which was calculated as [(experimental LDH−spontaneously released LDH)/(total LDH−spontaneously released LDH)]×100.

### Measurement of intracellular ATP.

T84 monolayers were grown in 48-well plates and inoculated as described above. Intracellular ATP levels were determined using an ATP Bioluminescence Assay kit CLS II (Roche Molecular Biochemicals) according to the manufacturer's protocol. Luminescence (10 s integration) was measured using a Lumat LB 5907 luminometer (EG&G Berthold). A standard curve generated from known concentrations of ATP was used to calculate the ATP content of each sample. The cellular ATP content is expressed relative to the cell number.

### Mitochondrial transmembrane potential.

To visually assess the mitochondrial transmembrane potential of treated T84 monolayers, a JC-1 mitochondrial membrane potential detection kit (Cell Technology) was used according to the manufacturer's protocol. JC-1 is a cell permeant dye that aggregates within mitochondria of healthy cells and forms a red-fluorescent multimeric complex (Smiley *et al.*, 1991). Conversely, in cells that have a low mitochondrial transmembrane potential, the dye is dispersed within the cytoplasm and remains in its fluorescent green monomeric form. Briefly, T84 cells were grown to 90 % confluence on chamber slides and treated with *C. jejuni* as described above, with camptothecin (4 μg ml^−1^), or H_2_O_2_ (0.5 mM). After incubation (4 h), monolayers were rinsed three times with PBS, and then DMEM containing JC-1 reagent and Hoechst 33258 (1 μM, Molecular Probes; 100 μl per well) was added to each well and incubated at 37 °C, 5 % CO_2_ for 25 min. Monolayers were rinsed once with JC-1 assay buffer. Slides were visualized by epifluorescent microscopy.

### Western blot analysis.

T84 monolayers were grown in six-well plates and treated with *C. jejuni* as described above or with camptothecin (4 μg ml^−1^). After incubation, monolayers were rinsed twice with PBS containing 1 mM EGTA (Sigma) and protease inhibitor cocktail (25 μl ml^−1^; P 8340, Sigma). Proteins were extracted from the monolayers by boiling the samples for 10 min in sample buffer (0.125 M Tris/HCl pH 6.8, 4 % SDS, 20 %, v/v, glycerol, 1.44 M 2-mercaptoethanol; Sigma), and stored at −80 °C until use. Proteins were separated by 10 % SDS-PAGE and transferred onto a 0.45 μM nitrocellulose membrane (Bio-Rad). Membranes were blocked for 1 h in TBS containing 5 % skim milk and 0.1 % Tween 20 and then rinsed briefly in TBS containing Tween 20 (TTBS). Membranes were incubated for 1 h at room temperature in TTBS containing 2 % BSA, and mouse anti-PARP antibody (1/1500 dilution, Roche) or mouse anti-*α*-tubulin antibody (1/3000 dilution, Molecular Probes) as the internal control. Membranes were washed with TTBS and then incubated for 1 h at room temperature in TTBS containing 2 % BSA, and horseradish-peroxidase-conjugated anti-mouse IgG antibody (1 : 5000 dilution, Sigma). Membranes were washed with TTBS and visualized by ECL detection (Amersham Pharmacia). Band densities were determined by image analysis using AlphaEaseFC software, v3.2.1 (Alpha Inotech).

### Invasion and adherence assays.

T84 monolayers were grown in 48-well plates and inoculated as described above. After incubation, infected monolayers were rinsed three times with PBS. To assess adherence, monolayers were lysed with 0.1 % Triton X-100 in PBS for 10 min at room temperature on an orbital shaker. Following lysis, bacteria were enumerated by plating tenfold serial dilutions onto Karmali agar. Invasion was determined using a gentamicin protection assay. After incubation, infected monolayers were rinsed three times with PBS. Monolayers were then incubated for 3 h with fresh tissue culture medium containing gentamicin (500 μg ml^−1^) to kill extracellular bacteria. Following incubation, monolayers were rinsed, lysed, and bacteria were enumerated as for the adherence assay. A preliminary experiment was conducted to ensure that a bactericidal concentration of gentamicin was used for the invasion assay. *C. jejuni* 81-176 (7.5×10^7^ c.f.u.) treated for 3 h with 100, 250 and 500 μg gentamicin ml^−1^ resulted in the recovery of 960, 276 and 22 c.f.u., confirming previously reported values (Monteville *et al.*, 2003). Subsequent invasion assays were conducted with 500 μg gentamicin ml^−1^.

### Statistical analysis.

All statistical calculations were performed with GraphPad InStat v.3.06 software (GraphPad Software). For each experiment, repetitions were conduced on separate occasions. Data are expressed as the means±sem, and compared by one-way analysis of variance, followed by the Tukey–Kramer multiple comparison test. Regression analysis was performed using Pearson correlation analysis. Statistical significance was established at a *P* value of <0.05.

## RESULTS

### *C. jejuni*-induced enterocyte death is consistent with oncosis

The effect of *C. jejuni* on enterocyte morphology was initially examined by transmission electron microscopy. Six hours post-inoculation, *C. jejuni*-treated T84 enterocytes appeared degenerated and exhibited extensive cytoplasmic vacuolation (results not shown). A loss of interdigitation between adjacent cells, and detached cells, were often observed. Nuclei were barely distinguishable from the cytoplasm, and nuclear condensation or fragmentation was not evident. Remarkably, cells exhibiting classic apoptotic morphology (i.e. condensed and fragmented nuclei) were conspicuously absent in *C. jejuni*-treated monolayers (100 cells from each of five separate regions of the monolayer were observed per treatment). In contrast, control monolayers exhibited well-defined nuclei and interdigitation between adjoining cells. Occasionally, as expected, cells exhibiting classic apoptotic morphology were observed in the control-treated monolayers (∼5 % of cells observed).

**Fig. 1. f1:**
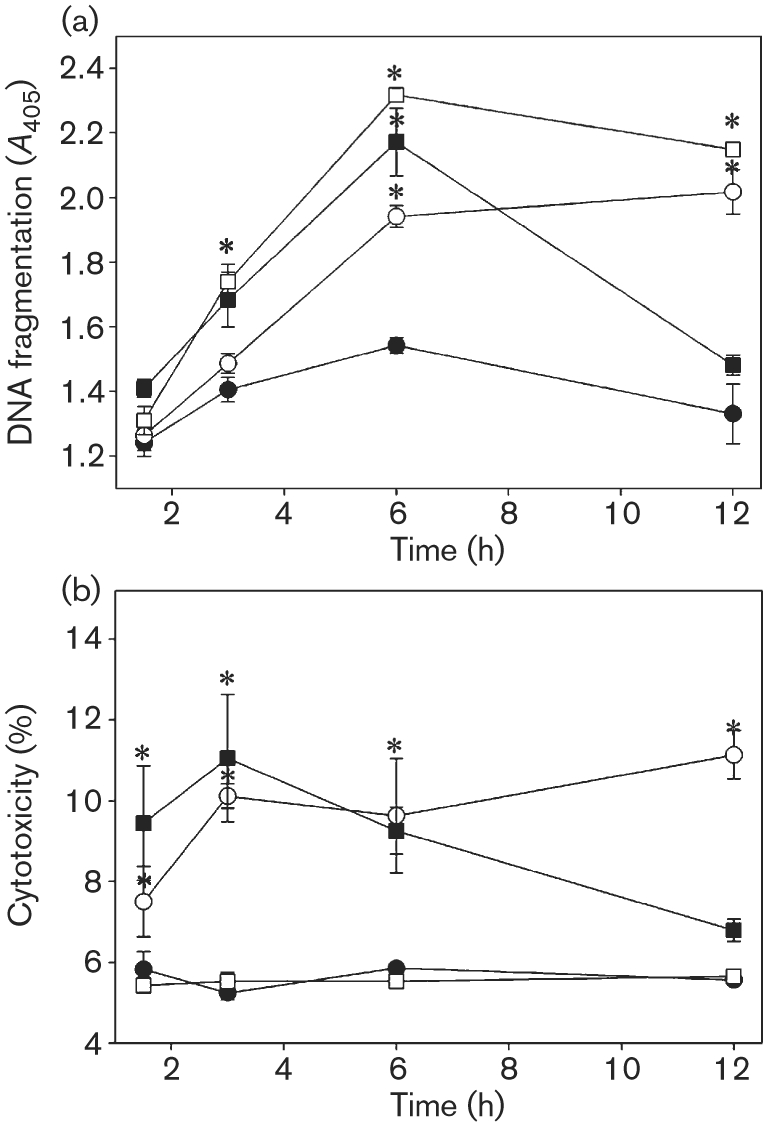
*C. jejuni* 81-176 induces rapid enterocyte cytotoxicity and oligonucleosomal DNA fragmentation. T84 monolayers were incubated with sterile growth medium (control, •), *C. jejuni* (○), H_2_O_2_ (▪) or camptothecin (□). Oligonucleosomal DNA fragmentation (a) and cytotoxicity (b) were determined at various times. Values are means±sem for three replicates (six observations per treatment). *, *P*<0.05 vs control treatment.

*C. jejuni* induced a time-dependent increase in oligonucleosomal DNA fragmentation that was significantly higher compared to that seen with the control treatment from 6 h onwards (Fig. 1a[Fig f1]). Monolayers treated with pro-apoptotic camptothecin also exhibited significant DNA fragmentation from 3 h onwards. Significant DNA fragmentation was exhibited in monolayers treated with pro-oncotic H_2_O_2_ at 3 and 6 h post-treatment. Oligonucleosomes were not detected by ELISA in lysates of *C. jejuni* in the absence of enterocytes, thus confirming that this assay was specific to epithelial cell DNA fragmentation (results not shown).

*C. jejuni*-treated monolayers exhibited significantly increased cytotoxicity from 3 h post-inoculation onwards, compared to the control treatment (Fig. 1b[Fig f1]). Monolayers treated with H_2_O_2_, an inducer of oncosis (Palomba *et al.*, 1999), exhibited significant cytotoxicity at 1.5 h (earliest time point measured). Cytotoxicity was not significantly different between the control and pro-apoptotic camptothecin treatments. In a preliminary study (in the absence of enterocytes) it was determined that *C. jejuni* does not possess detectable LDH activity of its own (results not shown).

**Fig. 2. f2:**
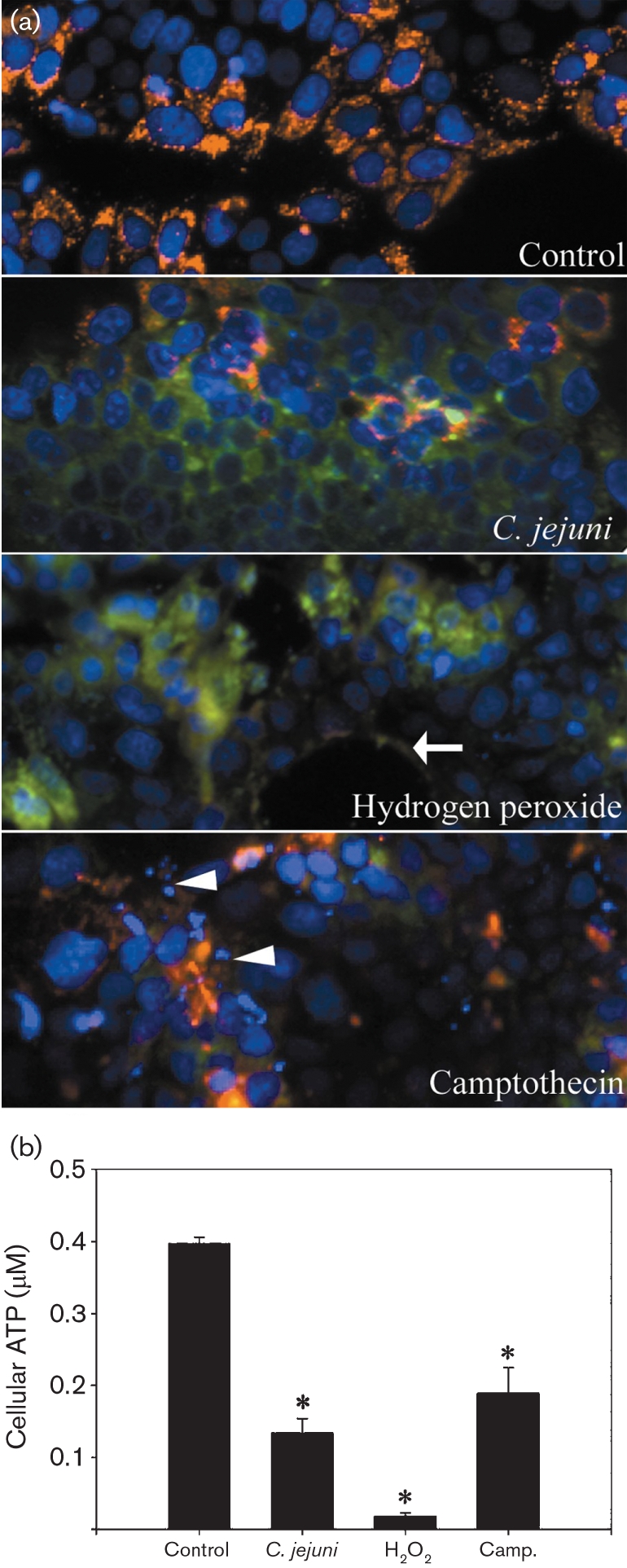
*C. jejuni* 81-176 causes mitochondrial transmembrane depolarization and depletion of intracellular ATP in enterocytes. (a) Representative images of mitochondrial transmembrane potential in T84 cells. T84 monolayers were treated for 6 h with sterile growth medium, *C. jejuni*, H_2_O_2_ or camptothecin and stained with JC-1. The red-fluorescent aggregated form of JC-1 indicates the presence of a high mitochondrial transmembrane potential, whereas cytoplasmic staining with the green fluorescent monomeric form of JC-1 indicates the loss of mitochondrial transmembrane potential. The arrow indicates where cells have sloughed off from the monolayer. Arrowheads indicate nuclear chromatin condensation and fragmentation. The figure shows representative images from one of two replicates. (b) Intracellular ATP levels of treated T84 monolayers were determined 6 h post-inoculation. Values are means±sem for three replicates (three observations per treatment). *, *P*<0.05 vs control treatment.

As cell death is largely regulated by mitochondria, experiments were conducted to determine the effect of *C. jejuni* on mitochondrial transmembrane potential. Aggregation of red-fluorescent JC-1 dye was observed in the mitochondria of the control-treated monolayers (Fig. 2a[Fig f2]). In contrast, *C. jejuni*-, H_2_O_2_-, and camptothecin-treated monolayers exhibited a diffuse green fluorescence, indicating the collapse of mitochondrial transmembrane potential. Green JC-1 staining was more intense in *C. jejuni*- and H_2_O_2_-treated cells, consistent with increased dye uptake as a result of the loss of plasma membrane integrity in these cells. As mitochondria serve as the major site for the generation of ATP, intracellular ATP was also measured in treated T84 monolayers. ATP levels were significantly reduced in *C. jejuni*-infected monolayers (6 h) compared to the control treatment (Fig. 2b[Fig f2]). Significantly decreased intracellular ATP levels were also observed in mononlayers treated with either H_2_O_2_ or campothecin compared to controls.

**Fig. 3. f3:**
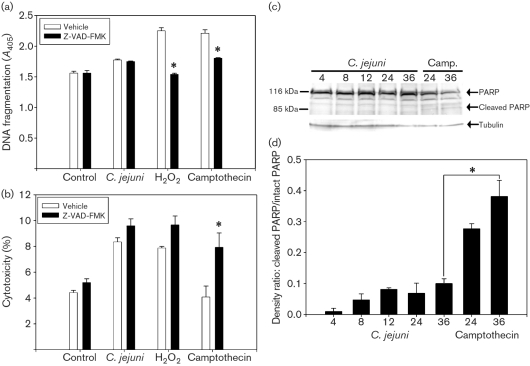
(a, b) *C. jejuni*-induced enterocyte death is caspase independent. T84 monolayers were inoculated with sterile growth medium, *C. jejuni* 81-176, H_2_O_2_ or camptothecin in the presence or absence of a pan-caspase inhibitor, Z-VAD-FMK. Oligonucleosomal DNA fragmentation (a) and cytotoxicity (b) were determined 6 h post-inoculation. Values are means±sem for three replicates (six observations per treatment). *, *P*<0.05 vs vehicle treatment. (c) PARP remains in its active, non-cleaved form in *C. jejuni*-treated T84 monolayers. T84 monolayers were incubated with *C. jejuni* 81-176 or pro-apoptotic camptothecin (control treatment) for various times and PARP was detected by Western blotting. Results were consistent across replicates and a representative blot from one of three replicates is shown. Western blots were quantified by densitometry (d); values are means±sem for three replicates. *, *P*<0.05 vs control treatment (36 h).

Since caspases mediate many aspects of cell death and inhibition of caspase-1 has been shown to prevent the *Salmonella*-induced oncosis (or ‘pyroptosis’) of macrophages (Brennan & Cookson, 2000), the involvement of caspases in *C. jejuni*-induced death was examined. Treatment of *C. jejuni*-infected monolayers with the pan-caspase inhibitor Z-VAD-FMK did not prevent oligonucleosomal DNA fragmentation (Fig. 3a[Fig f3]). In contrast, caspase inhibition prevented the DNA fragmentation induced by both pro-apoptotic camptothecin and pro-oncotic H_2_O_2_. Pan-caspase inhibition did not prevent *C. jejuni*-induced cytotoxicity (Fig. 3b[Fig f3]). Caspases also mediate the cleavage of PARP in apoptotic cells (Los *et al.*, 2002). In *C. jejuni*-treated T84 cells, PARP was present in its uncleaved state (116 kDa) and the PARP-cleavage fragment (89 kDa) was not detected throughout infection even up to 36 h post-inoculation (Fig. 3c[Fig f3]). In contrast, cleaved PARP was apparent in the camptothecin-treated monolayers, in which a time-dependent loss of the 116 kDa fragment was observed. Cleaved vs intact PARP ratios were significantly greater in camptothecin-treated cells (36 h) compared to values of monolayers exposed to *C. jejuni* for 36 h (Fig. 3d[Fig f3]).

### *C. jejuni* induces enterocyte death independent of CDT expression

**Fig. 4. f4:**
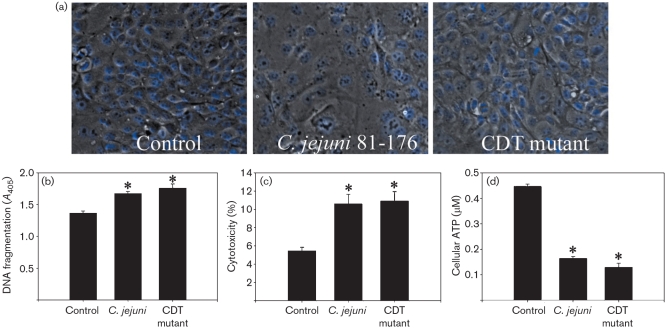
*C. jejuni* induces enterocyte death independent of CDT expression. (a) Characteristic distension of intestinal epithelial cells was observed in T84 cells treated with *C. jejuni* 81-176 but not with the isogenic CDT deletion mutant. Epithelial cells treated with sterile growth medium served as a negative control. A representative image (differential interference contrast) is shown from one of three replicates (three observations per treatment). Nuclei are stained blue. (b–d) T84 monolayers were incubated with sterile growth medium, *C. jejuni* or the CDT mutant and assayed for oligonucleosomal DNA fragmentation (b), cytotoxicity (c), or cellular ATP concentration (d). Values are means±sem for three replicates (three to six observations per treatment). *, *P*<0.05 vs control treatment.

The CDT deletion mutant was characterized to ensure that characteristic cytotoxic effects of the CDT were absent. PCR analysis of the CDT deletion mutant confirmed the absence of the *cdtB* gene and insertion of the Kn^R^ cassette (results not shown). PCR analysis also demonstrated the absence of plasmid DNA (used for construction of the mutant), confirming that the mutant was the product of a double homologous recombination event. After 30 h, T84 cells treated with the wild-type 81-176 displayed effects consistent with those previously described for CDT-intoxicated cells (Purdy *et al.*, 2000; Whitehouse *et al.*, 1998). Namely, cells were greatly distended (Fig. 4a[Fig f4]), there was a significant reduction in the number of cells undergoing mitosis, and cells displayed nuclear distension consistent with G2/M-phase cell cycle arrest (Table 1). Compared to the control treatment, there were significantly fewer cells per field of view corresponding to a combination of cell cycle arrest and increased oncosis. In contrast, T84 cells inoculated with the CDT mutant were not characteristically distended (Fig. 4a[Fig f4]) and the number of mitotic cells did not differ from the control treatment (Table 1). There were significantly fewer cells per field of view than for the control treatment, but significantly more cells for the wild-type *C. jejuni* treatment (i.e. corresponding to oncosis but not oncosis plus cell cycle arrest). Patches of the monolayer containing damaged or detached cells (‘death foci’) were also observed in both *C. jejuni*- and CDT mutant-treated monolayers.

The role of CDT in enterocyte oncosis was examined by comparing DNA fragmentation, cytotoxicity and ATP levels in T84 monolayers exposed to wild-type *C. jejuni* or the CDT mutant. In monolayers co-incubated for 6 h with the CDT mutant, both oligonucleosomal DNA fragmentation and cytotoxicity were significantly increased (Fig. 4b, c[Fig f4]) and cellular ATP was significantly reduced (Fig. 4d[Fig f4]) compared to the control treatment. Values did not differ significantly between the CDT mutant and wild-type *C. jejuni*. Deletion of the CDT gene from strain 81-176 also had no effect on T84 invasion (1.122±0.165 vs 1.143±0.158 % of the initial inoculum was internalized in T84 monolayers after 2 h of co-incubation with strain 81-176 and the CDT mutant respectively).

### *C. jejuni*-induced enterocyte oncosis is strain specific and is correlated with greater levels of host cell invasion

**Fig. 5. f5:**
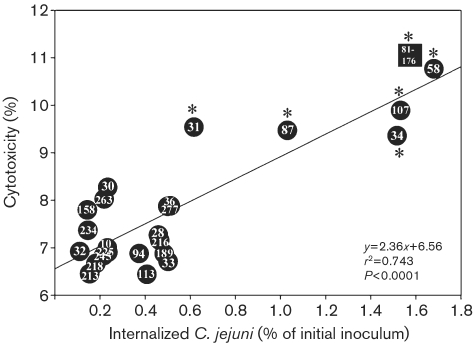
*C. jejuni*-induced cytotoxicity is correlated with higher levels of invasiveness. T84 monolayers were inoculated with strain 81-176 or one of 23 clinical *C. jejuni* isolates obtained from patients with enteritis (Chinook Health Region). Cytotoxicity and invasion were determined 3 h post-inoculation using the same monolayer. Each point represents the mean value of five replicates for each isolate (six observations). *, *P*<0.05 vs the negative control treatment for cytotoxicity.

To determine whether other strains of *C. jejuni* are capable of inducing oncosis, and investigate the relationship between cytotoxicity and cell invasion, T84 monolayers were inoculated with 23 clinical isolates of *C. jejuni*, and the pro-oncotic release of cytosolic LDH into the surrounding culture medium (‘cytotoxicity’) and the corresponding invasion of the cells were measured from the same monolayer. Five clinical isolates (CHR 31, 34, 58, 87, and 107) and strain 81-176 demonstrated significant cytotoxic LDH release from T84 cells within 3 h (Table 2). Cytotoxicity was significantly correlated with greater levels of host cell invasion (*y*=2.36*x*+6.56, *r*^2^=0.743, *P*<0.0001; Fig. 5). The *cdtB* gene was present in all clinical isolates except one (CHR 263) as determined by PCR analysis (not shown).

**Fig. 6. f6:**
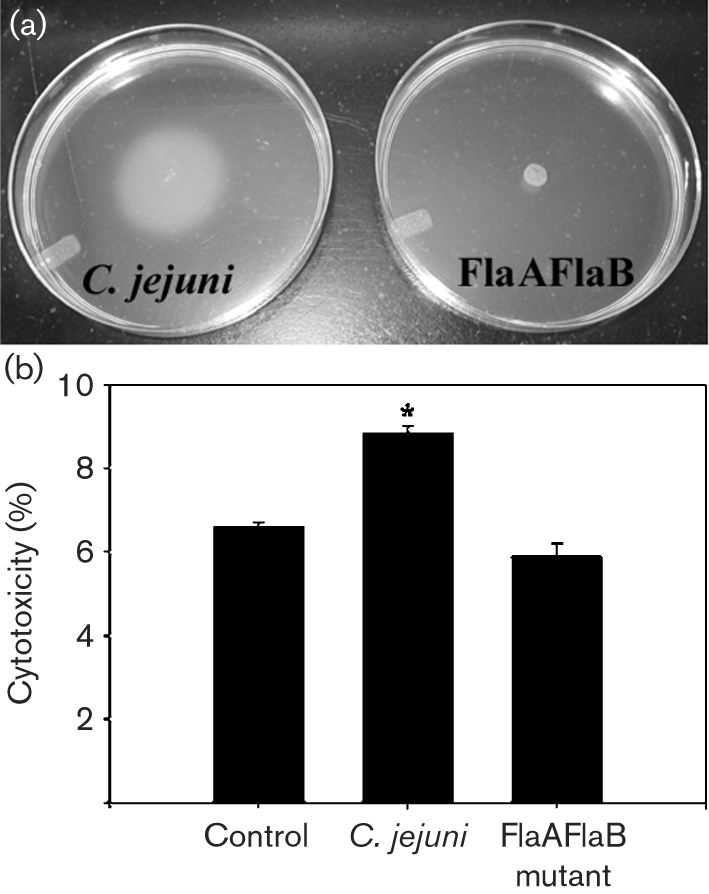
A non-adherent and non-invasive FlaAFlaB mutant of *C. jejuni* NCTC 11168 is defective in its ability to cause T84 enteroctyte oncosis. (a) The flagellar FlaAFlaB mutant is non-motile in soft agar (24 h). (b) Cytotoxicity is significantly increased in T84 monolayers treated with the wild-type strain NCTC 11168 compared to control- and FlaAFlaB mutant-treated monolayers (3 h post-inoculation). Each point represents the mean value of three replicates for each isolate (six observations). *, *P*<0.05 vs the negative control treatment for cytotoxicity.

To further address whether invasion plays a role in oncotic death, *C. jejuni* NCTC 11168 and an isogenic flagellar mutant were assessed for their ability to adhere and invade T84 enterocytes and cause cytotoxicity. The primers used to generate the mutant did not amplify the *flaA* and *flaB* genes from strain 81-176, necessitating the use of strain NCTC 11168 for generating a non-invasive mutant. The FlaAFlaB mutant was non-motile as determined by phase-contrast microscopy and swarming on motility agar (Fig. 6a[Fig f6]). Cytotoxicity, adhesion and invasion were determined 3 h post-inoculation using the same monolayer. Cytotoxicity was significantly increased in T84 monolayers treated with the wild-type strain NCTC 11168 compared to control- and FlaAFlaB mutant-treated monolayers (Fig. 6b[Fig f6]). Also, cytoxicity did not differ significantly between control- and FlaAFlaB mutant-treated monolayers. Compared to the wild-type strain, the FlaAFlaB mutant exhibited significantly decreased ability to adhere to T84 cells (11.20±0.61 vs 0.62±0.042 % of the initial inoculum adhered to T84 monolayers respectively). The FlaAFlaB mutant also exhibited significantly decreased ability to invade T84 cells (0.55±0.12 vs 0.0044±0.0010 % of the initial inoculum of the wild-type and FlaAFlaB mutant invaded T84 monolayers respectively). The *cdtB* gene was present in strain NCTC 11168 as determined by PCR.

## DISCUSSION

Enteric pathogens have evolved numerous strategies to circumvent the intestinal epithelial barrier and colonize their host. While several studies have described cytotoxic effects of *C. jejuni*, particularly the effects of its CDT, on cultured epithelial cells, the mode by which *C. jejuni* may cause enterocyte death has not been adequately characterized. Results of this study indicate, apparently for the first time, that *C. jejuni* induces enterocyte oncosis in a strain-dependent manner, and that death is correlated with increased invasiveness and does not require the presence of the CDT.

Previous studies demonstrated that *C. jejuni* induced apoptosis of monocytes and lymphocytes (Hickey *et al.*, 2005; Siegesmund *et al.*, 2004; Zhu *et al.*, 1999). In these studies, death occurred in a slow manner (>24 h) consistent with apoptosis. In contrast, we observed that *C. jejuni* induced rapid enterocyte death (3–6 h), inconsistent with apoptosis. Using a number of morphological and biochemical features to characterize the mode of cell death, we revealed that *C. jejuni*-treated enterocytes displayed features consistent with oncosis. *C. jejuni*-treated enterocytes exhibited cytoplasmic vacuolation, and nuclear condensation typical of apoptotic cells was not evident. Importantly, *C. jejuni* induced a rapid loss of plasma membrane integrity, which is a hallmark of oncosis. A similar rapid loss of plasma membrane integrity was previously noted in *C. jejuni*-treated HeLa epithelial cells (Newell & Pearson, 1984). Oncotic disruption of the epithelial plasma membrane would release cellular contents into the internal milieu, consistent with the acute inflammatory response observed in many cases of campylobacteriosis. However, further investigation is needed to determine the degree to which *C. jejuni*-induced enterocyte oncosis is involved in inflammatory diarrhoea.

Caspases mediate many aspects of apoptosis, including DNA fragmentation (Earnshaw *et al.*, 1999). While the morphological characteristics of *C. jejuni*-treated enterocytes were consistent with an oncotic death process, the cells also exhibited increased oligonucleosomal DNA fragmentation, a feature more typical of apoptosis. However, we also observed that *C. jejuni*-induced DNA fragmentation was independent of caspase activity, indicative of a non-apoptotic mode of death. Caspase-independent DNA fragmentation has been previously observed in epithelial cells treated with a mitochondrial uncoupler that induces oncosis (Dong *et al.*, 1997). In contrast, H_2_O_2_ induced caspase-dependent DNA cleavage. Although H_2_O_2_ is commonly used as an inducer of oncosis, it actually simultaneously causes both oncosis and apoptosis to varying degrees depending on the dose and extent of damage inflicted (Palomba *et al.*, 1999). Thus, it is not unexpected that H_2_O_2_ induced significant caspase-dependent DNA fragmentation in addition to its pro-oncotic effects. In camptothecin-treated cells, caspase inhibition prevented apoptosis (i.e. DNA fragmentation) as expected, whereas it potentiated oncosis (i.e. cytotoxicity), suggesting that death was not prevented but rather the mode of death was switched from apoptosis to oncosis. This is consistent with the notion that caspase inhibitors may promote alternative cell death pathways (i.e. oncosis or autophagy) (Vandenabeele *et al.*, 2006), and that oncosis functions as the default death pathway when other forms of cell death are blocked (Golstein & Kroemer, 2007). Indeed, previous studies have also observed that caspase inhibition induced cells to undergo oncosis despite treatment with an otherwise pro-apoptotic stimulus (Los *et al.*, 2002; Vandenabeele *et al.*, 2006).

Although caspases are primarily effectors of apoptotic death, caspase-1 has been shown to mediate *Salmonella*-induced macrophage oncosis (Brennan & Cookson, 2000). However, *C. jejuni*-induced enterocyte oncosis was not prevented by caspase inhibition, suggesting that oncosis occurs via a caspase-independent mechanism that is distinct from that of other pathogens. Notably, *C. jejuni* also induces caspase-1 and -9-independent apoptosis of macrophages (Siegesmund *et al.*, 2004).

We observed that *C. jejuni* caused disruption of the mitochondrial transmembrane potential and ATP depletion in association with its direct cytotoxic effects on enterocytes. Damaged mitochondria have been previously observed in cell culture and animal models of campylobacteriosis (De Melo *et al.*, 1989; Gao *et al.*, 1991; Humphrey *et al.*, 1986; Newell & Pearson, 1984), suggesting that *C. jejuni* may target mitochondria to induce enterocyte death. The targeting of mitochondria appears to be a common strategy used by many pathogens, including the closely related gastric pathogen *Helicobacter pylori* (Blanke, 2005). Mitochondria serve as executioners of both apoptotic and oncotic death (Kim *et al.*, 2003). In this study, treatment of enterocytes with camptothecin, H_2_O_2_ or *C. jejuni* caused mitochondrial transmembrane depolarization and ATP depletion. However, apoptotic features such as maintenance of plasma membrane integrity and PARP cleavage were only observed in camptothecin-treated cells. These results are consistent with the known mechanisms of action for these agents. Camptothecin causes apoptosis by inducing the release of proapoptotic factors from mitochondria, which ultimately leads to depolarization and ATP depletion (Kiechle & Zhang, 2002). H_2_O_2_ is a potent oxidant that very rapidly depolarizes the mitochondrial membrane and causes oncotic death. Mitochondrial injury is common to both modes of death, and hence may be involved in both *C. jejuni*-induced apoptosis of macrophages (Siegesmund *et al.*, 2004) and oncosis of enterocytes (this study). Importantly, mitochondrial injury may induce apoptosis if cellular ATP levels remain sufficient to complete early-stage ATP-dependent apoptotic events, but may induce oncosis if mitochondrial damage causes rapid and excessive ATP depletion (Eguchi *et al.*, 1997; Leist *et al.*, 1997). Thus, while ATP depletion may be evident in both apoptosis and oncosis, only oncosis arises as a consequence of ATP depletion (Kim *et al.*, 2003). Enterocytes are relatively active metabolically (Ma *et al.*, 2006), and thus may be particularly susceptible to the ATP-depleting effects of *C. jejuni*-induced mitochondrial injury and therefore exhibit rapid oncotic death. Interestingly, other enteric pathogens, including *Shigella flexneri* and pathogenic *E. coli*, also induce either oncosis or apoptosis depending on the cell line that is used (Fernandez-Prada *et al.*, 1998; Nonaka *et al.*, 2003).

The intrinsic apoptotic pathway is the best-characterized mechanism of mitochondria-dependent cell death. Activation of this pathway results in the formation of the apoptosome, which activates caspase-9 and the apoptotic death cascade (Li *et al.*, 1997). Results from our study demonstrate that *C. jejuni* induces enterocyte death via a caspase-independent mechanism. Intriguingly, caspase-9-independent mitochondrial dysfunction has also been implicated in *C. jejuni*-induced macrophage apoptosis, indicating that *C. jejuni* causes cell death via a unique mechanism that does not involve formation of the apoptosome (Siegesmund *et al.*, 2004). We are currently determining whether enterocyte death is mitochondria dependent and identifying the specific mediators involved.

During host infection, enterocytes are among the first cells that *C. jejuni* interacts with. The abilities of *C. jejuni* to invade and/or kill host cells via cytotoxins are thought to be the primary factors responsible for disease (Ketley, 1997; Wassenaar & Blaser, 1999). Interestingly, our findings indicate that *C. jejuni* invades enterocytes and causes oncosis by a mechanism that is independent of CDT, its only known toxin. The rapid nature of oncosis is not consistent with the slow apoptotic death typically associated with CDT intoxication (Purdy *et al.*, 2000; Whitehouse *et al.*, 1998). Also, we observed that enterocytes infected with the CDT mutant were killed to the same extent as those infected with wild-type *C. jejuni*. Most *Campylobacter* strains produce CDT (Bang *et al.*, 2001; Eyigor *et al.*, 1999; Pickett *et al.*, 1996), and we verified the presence of the *cdtB* gene in all but one of the clinical isolates that we examined. Consistent with a CDT-independent mechanism, oncosis was induced in a strain-dependent manner despite the ubiquitous presence of the *cdtB* gene. Previous studies have shown that *C. jejuni* is able to induce monocyte and lymphocyte apoptosis in a CDT-independent fashion (Siegesmund *et al.*, 2004; Zhu *et al.*, 1999). It is possible that *C. jejuni* produces other cytotoxins besides CDT, including shiga-like toxin and haemolysin (Wassenaar, 1997), although this is controversial, as it is not supported by genome sequence analysis of *C. jejuni* strains. Regardless of the factors involved, the results presented here establish that *C. jejuni* causes CDT-independent oncosis.

Previous studies have suggested that cell death may be associated with invasion. Loss of epithelial cell viability has been shown to coincide with *Campylobacter* invasion (Newell & Pearson, 1984). Furthermore, CDT-independent apoptosis was significantly reduced (∼50 %) in monocytes inoculated with an invasion-defective mutant (Siegesmund *et al.*, 2004). Similarly, we observed that the ability to cause oncosis was significantly decreased in a FlaAFlaB mutant (CDT^+^) that was defective in the ability to adhere to and invade enterocytes, suggesting that association with host cells (i.e. adherence and/or invasion) is required to induce cytotoxic effects. Our results also indicated that clinical isolates of *C. jejuni* induced enterocyte oncosis in a strain-specific manner, and that cytotoxicity was correlated with invasion. Lack of standardized assays for assessing cytotoxicity and invasion makes comparisons among reports difficult. Additionally, many of the studies pre-date the discovery of the CDT, so it is not always clear whether cytotoxicity was confounded by the CDT. Despite these difficulties, our observations are in agreement with previous studies by Klipstein *et al.* (1985, 1986), in which cytotoxicity was found principally among highly invasive clinical isolates. Noteworthy is their observation that strains isolated from patients in whom disease was severe also exhibited higher levels of invasiveness and cytotoxicity than those from patients with less severe enteritis. The lack of patient data for the clinical isolates that we used prevented us from verifying these observations, but other studies have failed to establish an association between cytotoxicity and invasion (Lindblom & Kaijser, 1995; Nadeau *et al.*, 2003), possibly reflecting the variable nature of *C. jejuni* strains and/or different methodologies used for assessment of cytotoxicity. Further investigation into the causal relationship between enterocyte invasion and oncosis by *C. jejuni* and the relevance to clinical disease is warranted.

In summary, findings from our study demonstrate that *C. jejuni* induces rapid oncotic, rather than apoptotic, enterocyte death. Our findings also demonstrate that oncosis is CDT independent, requires *C. jejuni–*enterocyte association, is dependent on the strain of *C. jejuni* and correlates with increased invasion ability. These observations offer insight into the pathogenesis of this important enteric pathogen, as enterocyte oncosis and subsequent epithelial malfunction may contribute to the intense inflammation often associated with campylobacteriosis. Future investigations of *C. jejuni*–enterocyte interaction will elucidate the molecular mechanisms of *C. jejuni*-induced oncosis and assess its contribution to *Campylobacter*-induced enteritis.

## Figures and Tables

**Table 1. t1:** Comparison of cytotoxic effects of wild-type *C. jejuni* 81-176 and the isogenic CDT mutant Semi-confluent T84 cells were grown on chamber slides and inoculated with *C. jejuni* 81-176 and the CDT mutant at a m.o.i. of 10 and incubated for 30 h. T84 cells inoculated with sterile growth medium served as a negative control. Microscopy and image analysis were performed as outlined in Methods. Results are means±sem for three replicates (three observations per treatment).

	**T84 cells per field of view**	**Average area of nucleus (μm^2^)**	**Mitotic cells per field of view**	**Death foci per field of view**
Neg. control	1144±40</sc>	3.92±0.23	12.7±3.2	0±0
81-176	660±49*	4.69±0.22	0.7±0.3*	5.3±1.5*
CDT mutant	865±75*†	4.04±0.28	7.0±1.0†	5.6±1.5*

*P<0.05 vs negative control treatment. †*P*<0.05 vs *C. jejuni* 81-176.

**Table 2. t2:** *C. jejuni*-induced cytotoxicity and invasion of T84 monolayers Confluent T84 monolayers were inoculated with strain 81-176 or one of 23 clinical *C. jejuni* isolates obtained from patients with enteritis (Chinook Health Region). Cytotoxicity and invasion were determined 3 h post-inoculation using the same monolayer. Values are the mean±sem of five replicates for each isolate (six observations). Only half of the isolates were assayed at one time (each time contained a control treatment and strain 81-176 (=reference). Values for the control and 81-176 did not differ between times.

**Treatment**	**Cytotoxicity (%)**	**Control cytotoxicity (%)**	**Internalized *C. jejuni* (% of initial inoculum)**
CHR 10	7.04±0.99	5.37±0.30	0.25±0.11*
CHR 28	7.31±0.41	5.21±0.71	0.48±0.17*
CHR 30	8.31±0.54	5.37±0.30	0.25±0.03*
CHR 31	9.58±0.98†	5.37±0.30	0.64±0.14*
CHR 32	6.96±0.57	5.37±0.30	0.13±0.06*
CHR 33	6.75±0.45	5.21±0.71	0.52±0.08*
CHR 34	9.40±0.91†	5.37±0.30	1.54±0.52
CHR 36	7.91±0.35	5.21±0.71	0.52±0.10*
CHR 58	10.81±0.88†	5.21±0.71	1.70±0.23
CHR 87	9.51±1.41†	5.37±0.30	1.05±0.27
CHR 94	6.92±1.07	5.21±0.71	0.39±0.16*
CHR 107	9.93±0.97†	5.21±0.71	1.55±0.08
CHR 113	6.48±0.36	5.21±0.71	0.43±0.18*
CHR 158	7.84±0.72	5.37±0.30	0.16±0.04*
CHR 189	6.91±0.25	5.21±0.71	0.50±0.03*
CHR 213	6.50±0.69	5.37±0.30	0.17±0.05*
CHR 216	7.19±0.65	5.21±0.71	0.48±0.09*
CHR 218	6.71±0.20	5.21±0.71	0.20±0.08*
CHR 225	6.95±0.38	5.21±0.71	0.26±0.08*
CHR 234	7.41±0.20	5.37±0.30	0.17±0.02*
CHR 245	6.88±0.59	5.37±0.30	0.24±0.07*
CHR 263	8.07±0.38	5.37±0.30	0.24±0.09*
CHR 277	7.92±0.58	5.37±0.30	0.53±0.09*
81-176	11.09±0.70†	5.47±0.51	1.59±0.07

*P<0.05 vs strain 81-176. †*P*<0.05 vs control treatment.

## Abbreviations

- CDT, cytolethal distending toxin
- LDH, lactate dehydrogenase
- PARP, poly(ADP-ribose) polymerase
